# The existential fracture model: reconceptualizing narcissistic personality disorder through a phenomenological-existential lens

**DOI:** 10.3389/fpsyt.2026.1771661

**Published:** 2026-02-16

**Authors:** Jian Sun

**Affiliations:** School of Educational Sciences, Guangxi Minzu Normal University, Chongzuo, China

**Keywords:** boundary situations, death anxiety, existential fracture, narcissistic personality disorder (NPD), ontological security, phenomenological psychopathology, self-grasping

## Abstract

Current paradigms for understanding Narcissistic Personality Disorder (NPD)—including psychoanalytic, cognitive-behavioral, and neuroscientific approaches—offer valuable insights but remain fragmented. They largely fail to explain the deep motivational structure of narcissism, particularly the co-existence of grandiose and vulnerable subtypes and the disorder’s profound resistance to treatment. This paper proposes an integrative meta-theoretical framework, the Existential Fracture model, to re-conceptualize NPD not merely as a cluster of symptoms but as a fundamental crisis of meaning arising from a systematic dissociation between primary lived experience and secondary symbolic representation. Drawing on Jaspers’ phenomenological psychopathology and Heidegger’s existential analytics, the model posits that narcissistic pathology originates from a collapse of ontological security and the individual’s world-picture (Weltbild). In response to existential “boundary situations” (Grenzsituationen), such as the awareness of mortality, the individual constructs a rigid, performative “idealized self” as a defensive fantasy of special exemption. This paper further integrates the Buddhist critique of “self-grasping” (atma-graha) to illuminate the narcissistic attachment to a fictional self-entity and the instrumentalization of others. The analysis reveals that both grandiose and vulnerable narcissism represent divergent defensive strategies against the same core existential anxieties, rooted in a fractured relationship with time, death, and intersubjectivity. The clinical implications call for a paradigm shift from symptom correction to ontological transformation. Therapeutic practices informed by phenomenological reduction, death awareness meditation, and mindful non-attachment are proposed to facilitate the move from a performative existence to an authentic one by rebuilding the capacity for symbolic integration of primary experience. This theoretical synthesis provides a robust platform for future research and humane clinical intervention, bridging subjective meaning-making with objective clinical observation.

## 1 Introduction

Narcissistic Personality Disorder (NPD) is a major diagnostic category within the DSM-5 classification of personality disorders, characterized by grandiosity, a need for admiration, and a marked deficit in empathy (1). While this descriptive diagnostic system demonstrates high clinical reliability, it possesses fundamental limitations in explaining the underlying dynamic mechanisms of narcissistic personality. As noted by Cain et al. (2), the current diagnostic criteria fail to adequately differentiate the qualitative distinctions between adaptive and pathological narcissism, nor do they elucidate the ontological roots of the collapse within the narcissist’s internal meaning system. Crucially, the DSM framework reduces narcissism to a collection of overt behavioral symptoms, overlooking its psychopathological essence as a rupture in the “self-world” relationship at an ontological level. This oversight is compounded by the model’s failure to adequately explain the distinction between grandiose and vulnerable manifestations of NPD—a distinction empirically established in contemporary personality psychology (e.g., 3). While often treated as stable subtypes, an alternative perspective, more aligned with the humanistic-existential tradition, views these as dynamic defensive positions that an individual may oscillate between, reflecting different strategies to manage the same core existential anxiety (4). This oversight often confines therapeutic interventions to behavioral modification, making fundamental structural change difficult to achieve.

Current research on narcissism is marked by significant theoretical fragmentation. The psychoanalytic tradition (5, 6), while profoundly revealing the causal links between early attachment trauma and narcissistic defenses, falls into the pitfall of childhood determinism. It cannot sufficiently explain why similar traumatic experiences lead to markedly different personality manifestations—such as grandiose or vulnerable narcissism—or account for other nuanced heterogeneities within narcissism (3). The cognitive-behavioral approach (7, 8) conceptualizes narcissism as a product of distorted core beliefs, emphasizing symptom alleviation through cognitive restructuring. However, this paradigm underestimates the driving force of unconscious motivations, and its intervention logic of “correcting cognition” fundamentally conflicts with the narcissist’s defensive fixation on their meaning structures. Meanwhile, the neuroscientific approach has identified biomarkers such as overactivation in the dorsolateral prefrontal cortex (DLPFC) and aberrant amygdala functional connectivity (9, 10), yet it reduces subjective experience to neurochemical events, thereby losing the depth of phenomenological understanding. In short, these three dominant paradigms operate in isolation. None have successfully established an integrative analytical framework that bridges “subjective meaning-making” with “objective biological foundations,” nor have they addressed the core theoretical puzzle of how grandiose and vulnerable narcissism can coexist within the same individual. Accordingly, the present approach departs from psychodynamic models that prioritize ego-structural repair, as well as from cognitive-deterministic approaches that frame pathology primarily in terms of distorted beliefs. Instead, it conceptualizes narcissistic suffering as a disturbance in the subject’s mode of being-in-the-world.

This study aims to propose a meta-theoretical framework—the Existential Fracture model—to reconceptualize NPD as a crisis of meaning resulting from a systematic dissociation between primary and secondary experience when an individual confronts boundary situations (Grenzsituationen). Drawing on the phenomenological psychopathological tradition of Jaspers (11), this model views narcissistic symptoms as defensive reconstructions following the collapse of the subject’s world-picture (Weltbild). Here, “world-picture” (Weltbild) refers to the pre-reflective yet symbolically mediated framework through which the subject experiences meaning, temporality, embodiment, and intersubjectivity as coherent and livable. Simultaneously, it incorporates a Heideggerian ontology of death anxiety, framing the grandiose self-fantasy as a delusional exemption from mortality. Furthermore, in a novel theoretical integration, the model incorporates the critique of “self-grasping” (ātma-grāha) from Eastern Zen Buddhism. This reveals that the narcissist is not only attached to a fictional, permanent self (self-grasping) but also dogmatically insists on control mechanisms that project their own rules onto the world (dharma-grasping). This dual attachment constitutes the fundamental obstacle preventing the narcissist from experiencing existential joy. By integrating three perspectives—phenomenological description, ontological interpretation, and cross-cultural critique—this study seeks to construct a unified theoretical platform that can both explain the deep dynamics of narcissism and guide innovative clinical interventions, thereby offering a new pathway for a human-scientific approach to personality disorder research.

This manuscript is explicitly positioned within the humanistic-existential tradition, which understands the human being not as a fixed egoic structure to be repaired or corrected, but as an open and situated possibility of experiencing existence. In this sense, the integration of phenomenology, existential philosophy, and Buddhist accounts of self-grasping aligns the present model with humanistic, existential, and transpersonal psychologies that emphasize the loosening or transcendence of egoic fixation, rather than its optimization or stabilization.

## 2 Literature review and theoretical framework

### 2.1 Multiple paradigms of narcissism and their limitations

The psychoanalytic school has laid the most substantial theoretical foundation for narcissism research. However, long-standing methodological tensions within it also constrain its capacity to fully explain contemporary narcissistic pathology. Classical Freudian theory defined narcissism as a regressive investment of libido into the ego, viewing it as a defensive posture where the individual withdraws from object-love and retreats to primary autoeroticism (12). Yet, this early model failed to adequately elucidate the paradox presented by narcissists in adulthood: the coexistence of complex social functioning and an internal sense of emptiness. Subsequent theorists expanded this framework along two paths: Kohut’s (5) deficit model emphasizes the lack of self-object experiences, proposing that persistent frustration of mirroring and idealization needs in childhood leads to fragmentation of the core self, forcing the individual to adopt grandiose omnipotence as a compensatory structure. In contrast, Kernberg’s (6) conflict model focuses on the pathological fusion of aggressive drives, arguing that a cold, derogatory rearing environment fosters a defensive self-structure that integrates a grandiose self with persecutory objects, centered on the repression of primal anxiety related to shame and envy.

Although both models offer insightful clinical observations, their shared presumption of “childhood determinism” constitutes a fundamental limitation. Firstly, both assume narcissistic pathology is a linear causal product of early relational patterns, struggling to explain why individuals with similar traumatic experiences may develop markedly different phenotypes—namely, the differentiation between grandiose and vulnerable narcissism (2, 13). While Kohut’s successors attempted to introduce the concept of “compensatory development,” they still did not resolve why some individuals adopt the aggressively expressive thick-skinned narcissism, while others become fixated on the shame-sensitive thin-skinned narcissism (14). More critically, this paradigm anchors narcissistic motivations entirely within the topology of object relations, almost entirely neglecting fundamental anxieties at the ontological level—particularly the primary fear of death, meaninglessness, and existential isolation. This isolation does not stem from a lack of interpersonal relationships but from the individual’s realization of an unbridgeable chasm between themselves and the world; even in intimate relationships, we ultimately face our own life experiences alone. When a narcissist proclaims, “I am unique and therefore should not be bound by ordinary rules,” the underlying discourse is not merely a distortion of object representations but a radical denial of finitude—a universal human condition. While psychoanalytic interpretation can trace the ontogeny of this denial, it fails to engage with its universal ontological structure and contemporary cultural specificity.

This deterministic stance also struggles to conceptualize grandiose and vulnerable narcissism as anything other than fixed characterological outcomes. In contrast, humanistic-existential approaches, by conceptualizing these manifestations as archetypal defensive positions (e.g., 4), offer a less deterministic framework. This view allows for greater clinical flexibility and acknowledges the individual’s existential agency in navigating these positions, rather than being rigidly determined by them. It is this more dynamic understanding of narcissistic phenomena that the present model seeks to advance.

Cognitive-Behavioral Therapy (CBT) and its derivative models (e.g., Young’s Schema Therapy) provide a clear technical roadmap for narcissism intervention. Beck’s cognitive theory attributes narcissistic symptoms to a core belief system of “unique superiority,” which maintains dysfunctional self-schemas through cognitive distortions like selective attention and magnifying inferences (7, 15). Young (8) further refined this into the “Self-Aggrandizer Schema Mode,” emphasizing how this mode inhibits the Vulnerable Child Mode to avoid the risk of exposing core emotional needs. These models offer significant operational advantages: they translate abstract personality traits into measurable, intervenable cognitive variables and achieve symptom alleviation through techniques like Socratic questioning and behavioral experiments.

However, the “rationalist bias” of the CBT paradigm renders it inadequate for explaining the tenacity and deep emotional core of narcissism. Its core hypothesis—that cognition is the antecedent cause of emotion and behavior—encounters a circular argument in narcissistic cases: when a therapist challenges the belief “I must be admired by everyone,” the narcissist is not merely holding an erroneous cognition; rather, the belief itself serves a more fundamental ontological function, namely, maintaining a symbolic protective shell that shields them from confronting the void. As Jaspers (11) cautioned, some psychic phenomena are not the result of “irrational thinking” but expressions of “comprehensible meaning”; they are “answers” to existential dilemmas, not “errors.” CBT’s treatment of narcissistic beliefs as “dysfunctional cognitions” to be corrected effectively reduces ontological struggles to epistemological fallacies. This critique finds a sobering echo at a broader disciplinary level: philosophy—the discipline often regarded as the paragon of rational inquiry—has its own practical modes even considered a manifestation of “disciplinary narcissism,” characterized by resistance to methodological consensus, a need to be perpetually correct, and intellectual posturing through complex terminology (16). If the very roots of rationality may be embedded in non-rational narcissistic soil, the limitations of a therapeutic paradigm relying solely on superficial rational techniques for shaking the existential foundations of narcissism become self-evident.

Furthermore, the paradigm’s systematic exclusion of “unconscious motivations” prevents it from explaining why narcissists might rationally acknowledge their problems yet emotionally be unable to relinquish grandiose fantasies. This “split between intellect and emotion” points precisely to more primitive self-preservative drives beneath the cognitive level. Terror Management Theory (TMT) research has confirmed that mortality salience strengthens an individual’s cultural worldview defense and self-esteem pursuit (17), and the narcissist’s grandiose self represents a pathological extreme of this defense. By failing to incorporate this existential driver into its theoretical core, CBT’s efficacy is often limited to the symptomatic surface, unable to disrupt the ontological foundations of the narcissistic structure.

Recent neuroimaging studies have provided important biological anchors for narcissism research. Meta-analyzes indicate that narcissistic traits are associated with overactivation of the dorsolateral prefrontal cortex (DLPFC), enhanced amygdala response to self-relevant threat stimuli, and aberrant functional connectivity within the default mode network (DMN) (9, 10, 18). These findings reveal neural inefficiencies in self-evaluation and emotion regulation among narcissists. Specifically, the cognitive dissonance between implicit self-deprecation (as seen in IAT) and explicit self-grandiosity may stem from decompensated conflict monitoring function in the anterior cingulate cortex (ACC). The strength of such research lies in its objectivity and replicability, providing physiological evidence for the “disease entity” of narcissism.

However, the “reductionist trap” of neuroscience leads to severe hermeneutic poverty. Firstly, neural correlates do not equal psychological causal mechanisms; discovering increased DLPFC activation only indicates this region’s involvement in narcissistic self-evaluation processes but cannot explain why the individual chooses to organize their self-experience around grandiosity rather than humility. Secondly, by objectifying subjective experience into brain activation patterns, this paradigm loses the irreducibility of the “first-person perspective” cherished by phenomenology. Jaspers (11) strongly criticized this “brain mythology”—equating mental life with neural processes is akin to reducing the meaning of a poem to the chemical composition of its ink. The subjective pain of the narcissist, such as feeling “existential loneliness in a crowd” or the “emptiness at the moment of praise,” cannot be exhausted by neuronal firing patterns. Finally, neuroscientific models lack a cultural dimension: they cannot explain why narcissism is expressed more often in vulnerable forms (with heightened shame and social anxiety) in collectivist cultures (e.g., East Asia), while grandiose manifestations are more prominent (flamboyance and competitiveness) in individualistic cultures. This cultural variability demands an interpretive framework that transcends purely biological explanations, which is precisely what the phenomenological-existential tradition offers.

### 2.2 Insights from the phenomenological-existential tradition

The phenomenological method established by Jaspers (11, 19) in *General Psychopathology* provides a key pathway to transcend the above limitations. He strictly distinguished between primary experience (*erste Ordnung*) and secondary experience (*zweite Ordnung*): the former is the immediately given, pre-reflective flow of life, the subject’s primordial encounter with the world in the “here and now”; the latter is the theoretical construction and symbolic narration of primary experience, a reflective organization by consciousness through language, causal categories, and value judgments. Primary experience thus refers to embodied, affective-perceptual experience, including visceral sensations, bodily tension or relaxation, affective tonality, and pre-reflective bodily orientation toward the world, prior to linguistic articulation or narrative interpretation. (e.g., sensations of emptiness in the chest, bodily contraction in response to shame, or diffuse somatic unease in the absence of explicit thought). Secondary experience thus operates primarily at the level of language, narrative identity, and meaning-making, enabling lived affect to be organized into communicable self-narratives, social roles, and moral evaluations.

In healthy subjects, primary and secondary experiences maintain a dynamic dialog—secondary experience lends intelligible form to life but remains open to revision by primary experience. The core pathology of the narcissist lies precisely in the rupture of this dialog: their primary experiences (e.g., existential emptiness when neglected) are defensively blocked from entering the symbolic system, while secondary experience rigidifies into a stagnant “idealized self-narrative,” creating a pathological blockage in what Ferrarello (20) describes as the “third-level intentionality structure.” In other words, narcissists cannot transform painful emotions into comprehensible symbolic representations and can only maintain “existential emptiness” through denial, dissociation, etc., ultimately leading to the instrumental rationalization of ethical relationships and the loss of an authentic state of being. This rupture is maintained through defensive operations such as dissociation, affective numbing, and somatic inhibition, whereby primary bodily experience is either split off or immediately neutralized before it can enter symbolic processing. When verbal symbolization is insufficient or prematurely foreclosed, alternative symbolic modalities—such as visual imagery, metaphor, cinematic identification, or other aesthetic forms—may phenomenologically function as transitional means for the indirect articulation of primary experience.

Jaspers further indicated that mental illness is essentially a manifestation of a broken meaning system (*Sinnzusammenhangsbruch*). When an individual’s world-picture (*Weltbilder*)—the cognitive-affective framework that integrates scattered experiences, establishes future expectations, and maintains identity—becomes systematically distorted, the subject falls into an inability to “understand” their own experiences (19). The narcissist’s grandiose fantasies should not be seen merely as symptoms but understood as “emergency patches” for their collapsed world-picture: when the true self cannot establish continuity through the caregiver’s mirroring, the individual must fabricate an “omnipotent self” to fill the identity void. Phenomenologically, this collapse of the world-picture manifests as a solidification of temporality—the narcissist is either immersed in a selectively remembered glorious past or fixated on a fantasized future exemption, losing any open attitude towards the “here and now.” The methodological insight from Jaspers is that researching narcissism must adopt an approach of understanding (*Verstehen*) rather than merely explaining (*Erklären*). The former employs empathy (*Einfühlung*) to enter the subject’s first-person perspective, revealing the internal logic of their world-picture; the latter remains at the level of third-party observational causal attribution. This distinction is crucial for narcissism research because the narcissist’s suffering lies precisely in the disjunction between their subjective experience and objective reality—behavioral frequencies or brain activation patterns alone cannot capture the paradoxical experience of “feeling profoundly existentially lonely even while surrounded by others.”

Heidegger’s (21) Daseinsanalytik provides a profound ontological framework for understanding narcissism. He described the human existential structure as “thrownness” (*Geworfenheit*). This thrownness gives rise to a fundamental “existential anxiety,” distinct from fear of a specific object, originating from two interrelated dimensions: First, the fatefulness of responsibility: While Dasein has the freedom to choose, it must bear absolute responsibility for its choices; this condition of “having-to-be-free” is a profound fate. Second, the groundlessness of meaning: The individual is thrown into a world full of contingency and finitude for no reason, where the values and meanings pursued lack any *a priori*, objective foundation as guarantee. For example, a child’s birth is not the result of their own choice but a determined, involuntary event. Beliefs such as “the soul precedes existence,” from Heidegger’s perspective, are precisely self-consolations constructed to escape this thrown nothingness, attempting to impose an illusory sense of control and purpose onto existence, yet remaining inauthentic illusions that evade Dasein’s authentic state.

Faced with the existential dilemma, a healthy response is the subject’s authentic (*Authenticity*) resoluteness: accepting life’s finitude and one’s own insignificance and sublimating the resulting existential anxiety into a creative life force, thereby living life’s density and fullness in “Being-towards-death” (22). This is a positive and constructive stance toward existence. In contrast, the narcissist is entrenched in an inauthentic (*inauthentic*) mode of being. Their pathological mechanism manifests as a twofold escape: On one hand, they unreflectively internalize the values of the “They” (*das Man*), such as pursuing status and wealth, to evade the burden of free choice, their deep-seated motive being a refusal to endow meaning upon their groundless life. On the other hand, they unconsciously alienate themselves into socially approved symbols, pursuing a symbolic immortality—for instance, vesting their self-worth entirely in fame, reputation, or posthumous memory—to construct an illusion of “immortality,” thereby avoiding the confrontation with the necessity of death (*mortality*). This is essentially a profound self-deception (*self-deception*).

However, an important cultural distinction must be made for this model. In the context of Eastern collectivist cultures, the pursuit of “securing a place in history” and intergenerational transmission, while superficially resembling the pursuit of “symbolic immortality,” is fundamentally different from pathological narcissism. This pursuit does not culminate in the ostentatious display of an atomized individual but rather entails the integration of the individual into the grand historical narrative of family, nation, or civilization as a means to realize self-worth. It provides the individual with a profound sense of meaning and purpose, its core being contribution and connection, rather than defense and exclusivity. Therefore, this path should be understood as an expression of “collective authenticity,” a healthy path provided by culture for individuals to come to terms with life and death and transcend finitude.

In contrast, pathological narcissistic immortality is referenced against the isolated, atomized individual and is exclusive and defensive in its means. This mode is pathological because its starting point is to transcend the nothingness of existence, but it actually intensifies the inner sense of emptiness. Its operational logic is as follows: To ward off existential anxiety, the narcissist constructs an idealized self. However, this idealized self is not an endogenous true self but a mirror self that must rely on external validation to be sustained. But external mirroring is inherently illusory and unstable: illusory because the mirror self is ultimately not the true self, merely a virtual image; unstable because the other’s mirroring is subject to the other’s needs, emotions, and other subjective factors—the other, as a mirror, is a constantly shifting and deforming surface. Consequently, the narcissist’s sense of self is placed in perpetual unease. Yet, because the narcissist mistakes the mirror self for the true self, fluctuations in this mirroring cause panic and rootlessness. To alleviate this anxiety, they must seek even more mirroring, thereby constructing increasingly rigid psychological defense systems. This, in turn, leads to deeper alienation from the true self, exacerbating fundamental loneliness and isolation, eventually plunging into a self-reinforcing vicious cycle.

Admittedly, from the perspective of Jacques Lacan’s mirror stage theory, the construction of the “self” begins with identification with an image (or the Other’s gaze) and is inherently fictional through “misrecognition.” In this sense, dependence on the mirror is a universal condition for the formation of human subjectivity. However, the key distinction between pathological narcissism and healthy personality lies precisely in the nature of the subject’s relationship with their own mirror image: healthy subjects can gradually become aware of the symbolic and fictional nature of self-identity, thereby anchoring their self-worth within broader relational networks and meaningful practices. The pathological narcissist, however, is addicted to this image; they absolutize the brilliant, complete illusion and attempt to make the entire real world conform to the maintenance of this virtual image. Therefore, while everyone’s self begins with the mirror; only the narcissist is imprisoned in an isolated cage that must be constantly polished and confirmed.

In summary, the narcissist’s grandiose delusion can be reinterpreted as a delusional denial of thrownness. Narcissists refuse to acknowledge their ontological finitude and existential contingency, instead constructing a narrative logic of “deserving special exemption.” This denial is not merely a cognitive bias but a total distortion of the mode of existence: they anchor their entire meaning system to a false self-image to avoid confronting the core anxieties of death, loneliness, and meaninglessness. Heidegger emphasized that inauthenticity is not a moral defect but a natural tendency of Dasein; but when this tendency solidifies into a rigid defensive structure to the extent that the individual completely loses the capacity for openness to primary experience, it can slide into the abyss of narcissism: the narcissist’s “Idealized Self” becomes what Heidegger calls concealment: it buries the truth of one’s own finitude once and for all, reducing every relationship to instrumental use and every choice to a calculation aimed at sustaining the illusion. The contribution of the existentialist perspective is that it reveals the cross-situational consistency of narcissism: whether manifested as aggressive grandiosity or shame-based vulnerability, both share the same ontological core—a fearful evasion of freedom and finitude.

The central role of death anxiety in narcissistic pathology receives empirical support and theoretical deepening through Terror Management Theory (TMT). Originating from Becker’s (23) cultural anthropological insights and systematized by Greenberg et al. (17), TMT’s basic hypothesis is that human self-awareness of mortality creates existential terror, which drives individuals to invest in a dual-defense system of cultural worldview and self-esteem. The cultural worldview is a shared symbolic system of meaning that provides symbolic immortality to buffer death anxiety; self-esteem is the perception of being a valuable individual according to the standards of the cultural script. Experiments confirm that mortality salience manipulations enhance cultural worldview defense and self-esteem striving, even influencing social judgments at a subliminal level (24).

However, the narcissist’s death defense has a fatal flaw; their grandiose self is a pathological extreme of this defense: they demand not just participation in the cultural meaning system but exclusive ownership of its symbolic resources, becoming a “deified exception.” The healthy individual’s worldview defense is transcendent—achieving symbolic immortality through connection to collective meaning networks while acknowledging their own ordinariness, forming a dialectical unity between the grand and the insignificant. The narcissist’s defense is monopolistic: their “special exemption” fantasy cannot be shared by the cultural community and must be built upon devaluing others. TMT research finds that highly narcissistic individuals show increased aggression and derogation of others after mortality salience (25), indicating a failure of social integration of their defense mechanisms. Furthermore, the fragility of the narcissistic defense stems from its detachment from social reality: the validity of a cultural worldview relies on social consensus, whereas the narcissist’s personal myth lacks intersubjective grounding, collapsing quickly upon encountering real-world setbacks and exposing the underlying death anxiety. This explains why narcissists erupt in “narcissistic rage” when challenged (26)—the threat is not merely to self-esteem but to the collapse of the symbolic system protecting them from death anxiety.

The integration of existentialism and TMT further reveals that the narcissist’s defense against death is not merely avoidant but distortive. They do not simply repress thoughts of death; instead, they construct a self-narrative of “transcending death”: by means of exaggerated achievements, boundless entitlement, and a belief in their own eternal impact, they fantasyally achieve physical immortality. This strategy differs from religious or artistic transcendence of death, which acknowledges death’s inevitability and creates meaning within that acknowledgment. The narcissist’s strategy denies death’s fundamental legitimacy, operating on the logic that “I am so special that death should not apply to me.” This delusional death denial prevents them from practicing what Heidegger advocated as “Being-towards-death”—focusing on the present and affirming authentic existence illuminated by the awareness of death. Instead, narcissists are either immersed in nostalgia or obsessed with future fantasies, unable to experience the authentic density of life in the present moment. Therefore, death anxiety is not only a trigger for narcissism but its organizing principle: the entire narcissistic structure revolves around defending against mortality. Symptomatic expressions like grandiosity, exploitation, and lack of empathy all serve this ontological aim. Grandiosity performs their specialness; Exploitation is necessary because they must use others’ feedback to sustain their self-image; Lack of Empathy exists because acknowledging others as independent subjects with their own desires would shatter the illusion of a world revolving around them. The fusion of the phenomenological-existential tradition with TMT provides a profound explanation for narcissism that transcends purely biological or descriptive models, anchoring symptoms, motivations, and fundamental existential dilemmas within a unified theoretical framework.

## 3 Core theoretical construction: narcissistic personality as existential fracture

### 3.1 The ontological mechanism of narcissism: from the fracture of the world-picture to defensive performance

The concept of ontological security (27), introduced in Modernity and Self-Identity, provides an anchor in existential sociology for understanding the origins of narcissism (27). Ontological security is not merely psychological safety but a deep, existential trust: a fundamental belief in the continuity of one’s life narrative, the reality of others as independent subjects, and the predictability of the physical and social environment. The establishment of this trust relies on consistent interactions with caregivers during infancy, with Erikson’s (28) “basic trust” forming its developmental psychological foundation (29). However, when the mirroring function from important others fails systematically—manifesting as unpredictable emotional responses, a self-centered organizational pattern, or a persistent disregard for the child’s authentic self-state—the child cannot construct self-continuity by internalizing stable object representations. The result is a fundamental existential insecurity: the individual not only feels abandoned by the world but essentially doubts “whether the self exists” and “whether the world is real.”

It is crucial to distinguish ontological insecurity, as employed in the present model, from developmental deficit or infantile determinist accounts. While the latter tend to conceptualize early relational failure as a lasting structural impairment that determines later personality organization, ontological insecurity refers to a destabilized mode of being-in-the-world that constrains, but does not abolish, existential possibility. Accordingly, early failures are not treated here as determining causes, but as historical conditions that shape the horizon within which existential choices are made. Even under conditions of ontological insecurity, the subject retains the capacity for existential choice, understood not as unrestricted freedom, but as the possibility of reorienting one’s relation to finitude, meaning, and the world.

Within this ontological fissure, the Idealized Self emerges as an emergency psychological construct. This construct is a response to the collapse of the individual’s world-picture (*Weltbild*)—the fundamental framework that confers meaning, temporality, embodiment, and intersubjectivity to experience. A stable world-picture allows for a fluid dialogue between primary experience and symbolic narration, enabling the individual to dwell authentically in the flow of time, to experience the body as a transparent medium of being, and to engage with others as independent subjects (*Mitsein*). In contrast, a collapsed world-picture, as seen in NPD, rigidifies this dynamic: meaning is replaced by existential absurdity, temporality fractures into a fixation on past glory or future exemption, embodied experience is defensively blocked, and others are reduced to instrumental objects for narcissistic supply. The narcissistic pathology is, therefore, a defensive response to this catastrophic loss of worldhood. Although this concept originates from Horney’s (30) theory of neurosis, it requires reinterpretation at an ontological level: the Idealized Self is not simply an inflated self-evaluation but a Performative Being self-generated by the subject when unable to rely on the external world for continuity of meaning. This performative being possesses three ontological characteristics:

First, self-referential closure. Its root lies in the caregiver’s failure to fulfill their function as a reliable “self-object” (5)—that is, as a decisive mirror and holding environment, they exhibit instability and inadequacy. Faced with this situation, acknowledging that one’s existential experience depends on such unreliable responses would mean confronting an immense, terrifying existential risk. To escape this vital terror, the individual adopts an extreme defensive strategy: completely denying any dependence on the unstable mirror, instead internalizing the mechanism for generating self-worth entirely within a closed psychological system. Although this choice implies loneliness, it provides a crucial sense of control. The core logic of this system’s operation is a self-referential circularity—”I am exceptional because I deem myself exceptional”—thereby rejecting any corrective feedback from real others to maintain its illusory stability and control.

Second, flight from the present (here and now). This flight manifests in both temporal and spatial dimensions: temporally, the performative being cannot reside within the continuous flow of “past-present-future,” but becomes fixated either on a reconstructed past or projected into a perpetual future; spatially, it cannot take root in the “here,” always attaching itself to a distant elsewhere. This spatio-temporal evasion stems from a fundamental conflict: whether reminiscing about the past, yearning for the future, or pinning hopes on a faraway place, these involve top-down subjective projections where the subject can maintain the illusion of omnipotent control. However, in the “here and now,” reality intrudes upon consciousness in a bottom-up manner, forcing the individual to inevitably confront their own finitude, vulnerability, and imperfection. Therefore, to sustain the illusion of the perfect self (idealized self), the individual can only persistently live within the fantasized construction of a “then” and “there,” evading the real, uncontrollable lived experience of the present.

Third, instrumentalization of the other. This stems from the narcissist’s inability to experience others as independent subjects engaged in “Being-with” (31), instead perceiving them solely as providers of mirroring or sources of threat. To maintain the operation of the performative being, the narcissist must reduce all interpersonal relationships to utilitarian exchanges of narcissistic supply (Kohut). The deep motive for this negation of the other’s independence (alterity) is that the core mechanism for maintaining the illusion of one’s own specialness and immortality precisely depends on continuous admiration and mirroring from others. However, acknowledging the other as an independent subject with their own will, needs, and world would shatter the narcissistic fantasy that “the world revolves around me,” causing the illusion of being the center of attention to collapse instantly. Confronting the reality shock of existential loneliness and ordinariness is highly threatening. To escape the primordial fear this evokes, the narcissist has no choice but to continuously instrumentalize others, fixing them into functional roles that serve their own psychological survival, thereby maintaining the self-centered fictional world they depend on.

This mechanism of instrumentalizing others to sustain narcissistic fantasy can be traced back to a survival strategy where the individual, after experiencing systematic devaluation, turns inward to compensate for the lack of social recognition (32). It is crucial to emphasize that this performative being corresponds precisely to the dual structure of narcissism revealed by contemporary personality psychology (33): agentic narcissism can be seen as offensive performance, actively displaying superiority to solicit external mirroring; whereas antagonistic narcissism constitutes defensive performance, protecting the fragile self by condemning others or playing the victim. This correspondence demonstrates that ontological-level analysis can provide a deep explanation for the different phenotypes observed in empirical psychology.

This mechanism can be further operationalized into a three-stage model of ontological security collapse: Stage 1 involves the erosion of basic trust, manifested in the persistent frustration of mirroring needs in infancy; Stage 2 is the fragmentation of the world-picture, where the child fails to form stable schemas of self, other, and world, leading to the collapse of the intelligibility (verständliche Zusammenhänge) of the meaning system (11); Stage 3 is the rigidification of the performative being, where the individual, to counter existential fragmentation, forcibly constructs an omnipotent, unquestionable idealized self, and maintains its internal consistency through strategies like persistent self-aggrandizement, devaluation of others, and external attribution.

This model underscores that the collapse of ontological security is not a deterministic outcome but a process that, at each stage, involves the individual’s tacit “choice” for defensive closure over openness, thereby preserving a notion of agency within the pathology. This model explains the self-reinforcing cycle of narcissistic defense: the foundation of the idealization is fictional, hence fragile; but precisely because it is fragile, it requires external validation; and the pathological craving for this external validation further weakens the capacity to internalize stable self-worth, creating what Giddens termed a “vicious cycle of existential anxiety.”

The three-level intentionality model of emotional processing, developed by Susana Ferrarello (20) based on Husserlian phenomenology, provides a micro-mechanism for precisely describing the symbolic rupture in narcissism. This model posits that emotional experience undergoes a three-level structuring from body to consciousness: Level 1 is embodied interest, the primary attentional orientation of the body to emotional stimuli, belonging to pre-reflective psycho-physiological integration; Level 2 is valuing, where reason makes meaning judgments about emotional stimuli, forming initial affective-cognitive connections; Level 3 is representation, where emotional content is transformed into an intelligible, communicable symbolic system, achieving intersubjective sharing.

In healthy subjects, these three levels form a dynamic continuum: Level 1 embodied experience smoothly enters Level 2 valuing and is then fluently transformed into Level 3 representation. Thus, the individual can both viscerally feel emotions and clearly articulate them, forming an “understandable self.”

However, in narcissists, a defensive representational block exists within this continuum. Its operational mechanism involves two steps: First, the narcissistic cognitive tendency assigns a highly negative and personalized Level 2 value to the Level 1 embodied interest—the initial emotional stimulus—for instance, directly interpreting external feedback as a threat and negation of their idealized self. Then, to avoid exposing the fictional nature of the self-structure, the narcissist must resort to psychological defenses such as denial, dissociation, or projection to arrest this already distorted value judgment at the pre-representational stage, thereby preventing it from reaching Level 3 consciousness where it could be reflected upon, communicated, or revised. The direct consequence of this block is a profound existential predicament: the individual can feel intense emotional arousal at the physiological level (Level 1) but cannot integrate it into a genuine, coherent self-experience, instead instantly experiencing it as the annihilation of self-worth (Level 2), and ultimately, due to the inability to symbolize it, falling into affective aphasia (Level 3). This state of “feeling without knowing” leads to a loss of experiential authenticity, trapping the narcissist for life within the prison of the “false self” described by Kohut (34).

### 3.2 Boundary situations and the differentiation of narcissistic defense strategies

Boundary situations (Grenzsituationen, i.e., boundary situations) are inevitable dilemmas in human life—extreme and adverse conditions that cannot be evaded through empirical experience or rational means, including death, suffering, conflict, guilt, and solitude, among others. Under normal circumstances, boundary situations can fulfill the following transcendent functions: First, they rupture the illusions of daily life, compelling individuals to confront the authenticity of existence; second, they prompt individuals to transcend mere intellect (Verstand) and activate deeper intuitive understanding (Vernunft), enabling self-awakening through reflection on these liminal experiences; third, they force individuals immersed in them to create meaning amid meaninglessness. However, for narcissists, the situation may be entirely different.

Boundary situations (Grenzsituationen), particularly the awareness of death, serve as the ultimate trigger for activating narcissistic defenses. Grandiose narcissists respond to this by constructing a “delusion of special dispensation”: “I deserve special treatment because I am extraordinary.” They groundlessly believe that their exceptional talents, achievements, or status should exempt them from the universal law of mortality. This fantasy is not merely a cognitive distortion but a comprehensive adjustment of their mode of being. Within the Terror Management Theory (TMT) framework, this fantasy can be seen as an extreme personalized form of cultural worldview defense—while healthy individuals achieve symbolic immortality by identifying with religious, national, or familial narratives, grandiose narcissists construct themselves as independent meaning systems, refusing attachment to any collective narrative due to their paranoid belief that external meaning systems cannot match their uniqueness. This mechanism can be decomposed into the following two parts:

First, a delusional reversal of causal logic. In the face of death, healthy defenses follow a “belonging-immortality” logic: “I belong to an eternal culture; therefore, I partake in immortality,” whereas grandiose narcissists execute a “uniqueness-exemption” logic: “I transcend culture, therefore I am exempt from death.” In the face of solitude, healthy defenses follow a “fusion-connection” logic: I belong to God, a nation, or humanity, thereby transcending individual boundaries to achieve connection, whereas grandiose narcissists execute a “uniqueness-transcendence” logic: their so-called interpersonal communication appears intersubjective but actually presupposes the deprivation of others’ subjectivity, essentially objectifying and reifying others, thereby maintaining a solipsistic false intersubjectivity in interpersonal interactions. This reversal of logic necessitates a cognitive investment in reality distortion: any evidence acknowledging their own limitations, such as aging, health decline, or competitive failure, is experienced as a fatal threat to their existential foundation, and thus must be immediately neutralized through aggression, denigration, or external attribution.

Second, a pathological escalation of social comparison. To sustain the uniqueness fantasy, grandiose narcissists must constantly seek downward comparison and uniqueness verification, which explains why they exhibit extreme competitiveness and derogatory behavior in peer evaluation tasks (3). Their behavior manifests in two aspects: at the level of secular achievements, grandiose narcissists exaggerate their own secular achievements, even if minor, while simultaneously belittling others’ secular achievements, even if world-renowned. When the secular level cannot provide sufficient support, they may retreat into religious or spiritual narratives, i.e., denying secular achievements altogether and claiming to be transcendent and spiritually elevated. However, this strategic retreat from secular comparison to a transcendent persona is actually a continuation of the same pathological structure in different contexts.

In contrast to the aggressive externalization of grandiose narcissism, vulnerable narcissists adopt a “victimhood narrative” as a defense strategy against boundary situations. This strategy appears humble on the surface but is actually a more covert and circuitous construction of moral privilege: by exaggerating the injustice and uniqueness of their suffering experiences, vulnerable narcissists position themselves for moral exemption—”I deserve special treatment because I have been victimized.” This mechanism incorporates an ethical dimension, transforming existential anxiety into ethical complaint, thereby avoiding direct confrontation with more fundamental dilemmas such as death or solitude.

The psychological structure of the victimhood narrative comprises three synergistic components:

First, the singularization of suffering. Vulnerable narcissists tend to experience their pain as an “absolute solitude that no one can understand.” This singularization exempts them from framing their suffering within the narrative framework of universal human suffering, thus avoiding empathy for others and diluting their moral capital.

Second, the projective externalization of responsibility. All negative experiences are attributed to external malice, such as others’ jealousy, societal injustice, or fateful manipulation. This external attribution not only protects their fragile self-core from the erosion of shame but also maintains their ontological innocence—if the pain of their thrownness is not self-inflicted, then they need not bear the absurd responsibility of existence.

Third, the reversal of moral status. By occupying the victim position, vulnerable narcissists recast interpersonal interactions into an “I deserve-you owe” power relationship. Their covert control strategies aim to compel others to provide narcissistic supply while maintaining their own moral superiority. For example, they rationalize emotional blackmail and passive aggression as defenses of justice rather than active harm to others.

This strategy manifests the paradox of thin-skinned narcissism: their subjective experience is full of shame and insecurity, but this shame is not a healthy moral emotion; it is narcissistic shame—stemming from self-attacks rooted in “I should be perfect but have failed.” Horney (30) described this as the hidden expansiveness of the “self-effacing” personality: through self-deprecation, individuals instead gain a反向 sense of grandiosity, i.e., precisely because I suffer so deeply, I am more profound than those麻木 ordinary people.

Although grandiose and vulnerable narcissism appear behaviorally opposite, they share an insistence on self-exceptionality (35). The coexistence of “thick-skinned” narcissism and “thin-skinned” narcissism within the same individual (36) further suggests an intrinsic consistency in their psychological structures. The core mechanism can be understood as follows: external excessive self-grandiosity often serves to conceal deep-seated feelings of powerlessness, shame, and self-inadequacy; while overt shyness and withdrawal may be used to hide a hidden sense of self-importance. The key distinction lies in the narrative frameworks they construct to rationalize their own “specialness”: the logic of grandiose narcissists is “I am exceptional, therefore I deserve special treatment”; whereas vulnerable narcissists follow the narrative logic of “I have been victimized, therefore I deserve special treatment.”

It is noteworthy that healthy individuals and narcissists exhibit fundamental differences in intentionality when facing boundary situations. This difference manifests essential disparities across the following four levels: At the level of motivational roots, the intentionality of healthy individuals is rooted in attachment needs, with a core motivation to be understood and witnessed, ultimately aiming to integrate traumatic experiences and rebuild psychological continuity and security. Their narratives are inward-directed, aimed at self-repair. In contrast, the intentionality of narcissists is rooted in narcissistic survival, with a core motivation to extract privilege and exemption. Their narratives are outward-directed, aimed at controlling others. At the level of emotional foundation, the primary emotion of healthy individuals is sadness, accompanied by a mourning process. Although painful, they allow themselves to be vulnerable and have the potential to move toward healing through connection. In contrast, the core emotions of narcissists are narcissistic shame and rage. Their “pain” is actually a defensive fear of their own vulnerability being exposed, quickly transforming into outwardly directed moral indignation. At the level of relational mode, healthy individuals desire intersubjective confirmation, i.e., that their pain can be seen and accepted by another independent subject. In contrast, narcissists seek instrumental submission; others are viewed as resources for providing “narcissistic supply,” and their independence is negated. Finally, when others offer empathetic understanding, healthy victims experience the realization of intersubjective connection; their core attachment needs are satisfied, thus feeling comforted. In contrast, narcissists do not need understanding but tribute. Therefore, they feel deprived and angry because their strategies for controlling others have failed.

### 3.3 Cross-cultural verification: the extreme pole of narcissism under Zen’s theory of self-grasping

Zen Buddhism, a major strand of Eastern philosophy, furnishes an external cultural lens for interrogating the pathological structure of narcissism. Its core teaching of anātman (“non-self”) strikes at the root error of the narcissist’s inflated self-importance: no permanent, independent “self” entity exists to be grasped. Zen praxis aims at dismantling self-grasping through a systematic deconstruction of the self, exposing its dependently-originated and empty nature. Narcissism, by contrast, channels all psychic energy into clinging to and magnifying the illusion of self. Between these two world-views lies a fundamental tension.

A brief ontological clarification is necessary at this point. The Buddhist conception of non-self (anātman) invoked here differs fundamentally from the ontologies implied in many theistic, soul-based, or salvation-oriented religious traditions. Zen does not posit an enduring metaphysical self, immortal soul, or transcendent entity to be redeemed or perfected. Rather, it offers a phenomenological-existential analysis of suffering grounded in impermanence, dependent origination, and the cessation of grasping. The critique of self-grasping therefore does not aim at replacing one metaphysical foundation with another, but at loosening attachment to any reified conception of being.

Within clinical theory, the self is usually taken as a more or less stable representational structure; Zen, however, reveals that its illusoriness and impermanence are far more radical. Zen traces all suffering to attachment and therefore instructs practitioners to release their grip on the phantom self and to realize the interconnectedness of all things (37). This fundamental denial of a separate, permanent agent constitutes a thoroughgoing transcendence of narcissistic modes of being. In detail, the three forms of self-grasping map directly onto (a) sense of entitlement, (b) compulsive rule-setting, and (c) instrumentalization of others.

First, the grasping to self (ātma-grāha) signifies that the individual is deluded by a fictitious self-entity, mistakenly believing that the body, thoughts, and other aggregates constitute a permanent and unchanging self. The state of self-grasping gives rise to fundamental negative emotions such as attachment, aversion, and ignorance. In narcissism, this manifests as the absolutization of entitlement. The narcissist not only mistakes the body, achievements, and status for the essence of the “self” but also demands that these experiential attributes be granted a special status at the ontological level. This corresponds to the rigidity of “attitudes” or “viewpoints” (Anschauungen) described by Jaspers (19): the narcissist reifies “I deserve special treatment” from a negotiable social expectation into an ontological truth. Any action challenging this truth is experienced as an attack on the very existence of the self.

Second, the grasping to phenomena (dharma-grāha) refers to the individual’s attachment to misperceiving all phenomena as truly existing objects, considering matter to be absolutely real and concepts to be absolute truths. The state of phenomena-grasping leads to rigid thinking and conceptual arrogance. In narcissism, this transforms into the authoritarian control of rules. The grandiose narcissist demands that others must adhere to the standards they establish, while the vulnerable narcissist imposes the moral law that “you are responsible for my suffering” onto others. The pathology of phenomena-grasping lies in the narcissist’s inability to perceive the socially constructed nature of rules; instead, they instrumentalize rules as extensions of the self. This represents an extreme form of the inauthenticity of the “they” (das Man) critiqued by Heidegger (31): the narcissist not only is lost in public opinion but also hijacks public opinion to serve self-deification. The difference from the “pathological grandiose self-structure” described by Kernberg (6) is that Kernberg emphasizes the distortion in the content of self-representations, whereas the Zen perspective reveals the grasping nature of the cognitive framework itself—even if the content of the rules changes, the attachment to the notion that “rules must serve me” remains unchanged. In other words, Kernberg emphasizes that the narcissist internally adheres to a narrative structure of a pathological grandiose self, while Zen explains that, regardless of how the specific content of the script is changed, the narcissist’s rigid narrative pattern of “there must be a system that serves me” remains constant.

Third, conceptual discrimination (vikalpa) — the extreme polarization of the subject-object dichotomy, which splits the world into “I” and “not-I.” In narcissism, this manifests as the complete instrumentalization of the Other. Lacking the courage and capacity to experience the ontological unity of “Being-with” (Mitsein), the narcissist must reify others into functional objects: either as mirroring providers who supply admiration, as sources of threat who need to be denigrated, or as moral judges who need to be persuaded. This process is not merely a pathologicalization of object relations (6), but an alienation at the ontological level: the subjectivity of the Other is perceived as a threat to the narcissist’s self-identity, because it may introduce uncontrollable variables that challenge the narcissist’s fragile self-narrative. This explains why narcissists in intimate relationships experience both craving and fear: craving stems from the need for supply, while fear arises because the independence of the Other can deconstruct the integrity of the fictional self.

It is necessary to make a special clarification from a cross-cultural perspective: the Zen critique of narcissism introduces a crucial hypothesis: collectivist Eastern cultures may suppress the expression of grandiose narcissism but reinforce vulnerable narcissism via the pathway of “relational self-grasping.” Hofstede’s (38) cultural dimensions theory points out that collectivist cultures emphasize harmony, face maintenance, and interdependence, making the direct expression of the self-aggrandizing strategies of grandiose narcissism socially too costly. However, the cultural value system itself does not eliminate self-grasping; it merely transforms its manifestations. Within an Eastern context, vulnerable narcissism may gain cultural adaptation advantages under the legitimate guise of a “relational self”: by emphasizing “my sacrifices for the family” or “my suffering stems from undertaking collective responsibilities,” individuals can repackage narcissistic needs as culturally endorsed senses of duty.

This cultural moderation effect can operate through the mediating mechanism of self-construal. An independent self-construal promotes grandiose narcissism (e.g., “I am unique, therefore I am proud”), whereas an interdependent self-construal may promote the victimhood narrative of vulnerable narcissism (e.g., “I sacrificed for the relationship, therefore I deserve compensation”). This interpretation is consistent with cross-cultural empirical findings indicating that collectivist cultural contexts are associated with lower levels of overt grandiose narcissism but relatively higher levels of covert or vulnerable narcissistic features, particularly when mediated by interdependent self-construal (e.g., 39–41).

The theoretical significance of this cross-cultural integration is that it transcends simplistic “cultural relativism”—narcissism is not an arbitrary product of cultural construction but rather the optimal adaptive expression of the structure of self-grasping under cultural constraints. Ironically, the Zen doctrine of “non-self” (anātman), which in Eastern cultures should inherently act to inhibit narcissism, can itself be distorted into another tool for narcissism if its essence of emptiness is misunderstood—”Because I have attained the state of non-self, I am superior to ordinary people who are still attached to a self.” This reveals the complex interplay between cultural values and deep psychological structures: culture may suppress the overt symptoms of narcissism, yet it may also provide moral legitimacy for its covert forms, while the true pathological root—the attachment to a self-entity—continues to operate beneath the cultural surface.

## 4 Clinical and philosophical implications: from symptom modification to ontological transformation

### 4.1 A paradigm shift in therapeutic philosophy: the ontological practice of reconstructing the world picture

Traditional psychotherapy views narcissism as a dysfunction requiring correction, operating on the logical premise of a pre-existing “normal” psychological structure. However, grounded in a Jaspersian phenomenological-existential framework, the healing of narcissism is not a return to some transcendental norm, but rather the reconstruction of a collapsed world-picture (Weltbild), allowing primary experiences to re-enter the process of symbolization, thereby restoring the individual’s authentic connection with the world. This paradigm shift signifies an elevation of the therapeutic goal from “symptom elimination” to “transformation of the mode of existence.” The core lies in assisting the narcissist to complete the leap from performative being to authentic existence. This leap cannot be achieved through simple cognitive restructuring, as cognition itself has been co-opted as a defensive tool within the narcissistic structure; genuine transformation must occur at a pre-cognitive level, reshaping the individual’s fundamental stance toward how they live in time, how they coexist with others, and how they respond to death.

Phenomenological reduction, as a core therapeutic technique, is not equivalent to “thought recording” in Cognitive Behavioral Therapy. Instead, it guides the narcissist to suspend (epoché) their natural faith in the idealized self. From the natural attitude, the narcissist takes “I must be special” as a given of the world. Phenomenological reduction training, however, requires the client to treat the belief “I must be special” itself as an object of experience. The aim is not to logically challenge the belief’s validity, as in cognitive-behavioral approaches—a tactic that often fails because the client, even while rationally acknowledging its absurdity, may still compulsively adhere to it. The task is to assist the client in becoming aware of the primary lack that this intentional structure represents, for only by doing so can the existential emptiness of the narcissistic defense be exposed. It is reasonable to believe that if the client can become aware that their pursuit of excellence, immortality, and specialness is essentially a symbolic substitute for the primary anxiety of “Do I truly exist?”,”, then the idealized self ceases to be an oppressive imperative and is de-reified into a series of fluid experiences, thereby making space for the generation of new meaning.

How, then, can we help the narcissist become aware of the primary lack underlying their narcissism? The powerful defenses constructed by the narcissist make this step exceedingly difficult, but not impossible. I posit that the following paths may help achieve this goal:

The first path is Death Meditation. The awareness of death is both the core trauma and the pivotal point of transformation in narcissistic pathology. Traditional psychotherapy often avoids the topic of death or pathologizes it as “death anxiety” to be eliminated. From an existential perspective, however, Death Awareness Meditation is not a negative exercise in terrifying imagination, but an active practice of Heidegger’s “Being-towards-death” (Sein zum Tode). The essential point is to guide the client, within the safety of the therapeutic relationship, to systematically contemplate the finitude of their own life—death is not merely a static event awaiting at the end of life, but an ongoing process of perishing intrinsic to every moment of life. Just as the blossoming of life is a process, death is not an abrupt cessation but a gradual dissipation permeating existence. Realizing that we do not suddenly die at a certain moment, but are simultaneously experiencing life and death at every moment, can prompt a fundamental awakening. Clinically, this work unfolds across intertwined levels of bodily lived experience (e.g., visceral sensations of finitude), affective regulation (e.g., tolerating panic, grief, or emptiness without immediate defensive enactment), and symbolic elaboration (e.g., allowing new meanings of existence to emerge without premature narrative closure). It is important to emphasize that death-related work, as discussed here, is not conceived as a standardized technique or an early-stage intervention. Rather, it represents a later-phase, carefully titrated existential exploration, conducted only when sufficient therapeutic alliance, affect tolerance, and relational safety have been established.

Experiencing death as a process is subversive for the narcissist because it deconstructs the ontological foundation of “special exemption.” When death is authentically and continuously apprehended as “my own, non-transferable possibility” (31), any fantasy based on “I should be immortal” loses its power of self-deception. It is reasonable to believe that sustained death meditation may initially intensify the narcissist’s existential panic—their performative self teeters as it loses the support of future projection, potentially manifesting as intense aggression or therapy dropout. At this stage, bodily defenses may manifest as dissociation, muscular rigidity, compulsive speech, or sudden withdrawal, and the therapist’s task is not to push through these reactions but to slow the process, restore grounding, and re-establish a sense of embodied safety. However, if they can endure this “night of the limit,” the client may experience the first glimmer of existential joy: when the burden of perpetual performance is lifted, primary experience can briefly manifest in the “present moment,” and a sense of “I am” that requires no justification quietly emerges. This experience, though faint, holds immense transformative potential, as it provides an alternative source of meaning—no longer derived from “I am better than others,” but from “I am authentically experiencing my existence.”

The second path is Mindful De-grasping. The Zen tradition of “seeing one’s nature and realizing one’s essence” offers a non-cognitive path for transforming narcissism. Contemporary psychology operationalizes “mindfulness” as a cultivatable trait—the ability to maintain non-judgmental attention to present-moment experience (42). Its core dimensions include observing, describing, acting with awareness, non-judging, and non-reacting (43). The mindful de-grasping discussed here is not merely attentional training but aims to systematically loosen the rigid self-structure upon which narcissism relies. This process can be elucidated through two complementary theoretical perspectives: the Zen contemplation of “No-Self” (anātman), and the phenomenological suspension of the Lacanian “mirror self.” Importantly, the bracketing of the mirror experience does not imply the disappearance of the Other in a relational sense. Rather, the therapist remains present as a non-evaluative, non-mirroring Other, offering a stable relational ground without reinforcing performative selfhood.

On the one hand, based on the Zen contemplation of “No-Self,” mindfulness directly addresses the core of self-grasping (ātma-grāha). It guides the narcissist to observe the dependent origination (pratītyasamutpāda) of the “self”: at the point where each self-narrative arises, to inquire, “From what conditions (the five aggregates, skandhas) is this ‘I’ constituted?” Sustained observation can expose the emptiness (śūnyatā) of self-perception, revealing that the so-called constant self is merely an illusory aggregation of momentary phenomena. Regarding phenomena-grasping (dharma-grāha), mindfulness training focuses on the groundlessness of rules and expectations, weakening the cognitive rigidity with which the narcissist projects internal needs as objective laws. Simultaneously, it cultivates awareness of being-with, helping the individual transcend the discriminating duality that instrumentalizes others.

On the other hand, from the perspectives of phenomenology and Lacanian theory, the healing mechanism of mindfulness lies in its suspension of the “mirror self.” As discussed earlier, Lacan revealed that the construction of the self begins with identification with the mirror image, inherently carrying the fiction of “misrecognition” (méconnaissance). The narcissist is utterly fixated on this image, living in a theater constructed by the gaze of the Other. The revolutionary aspect of the “non-judgmental present-moment awareness” advocated by mindfulness is that it withdraws attention from the fixation on “who I am” (a defined, solidified object) and anchors it in the pure process of “I am existing.” This is, in practice, stepping out of the mirror theater and returning to the prereflective, direct stream of experience, thereby repairing the rupture with the authentic experience of being. Therefore, mindfulness not only dismantles the “self-grasping” of Zen but also loosens the “mirror identification” of Lacan, offering the possibility of rebuilding a fluid, open sense of self rooted in lived experience.

However, one must be vigilant against the clinical risk of mindfulness practices being instrumentalized by the narcissist—turning the practice itself into new capital for “I am superior.” Research has found that mind-body practices like mindfulness meditation may not ameliorate narcissism but can instead lead to higher levels of self-enhancement, explaining this risk of instrumentalization (44). To avoid this pitfall, the therapist must lead by example, embodying a mode of being without self. If the therapist remains entangled in self-grasping, their “teaching” of mindfulness becomes a new performance within a power dynamic. Thus, Mindful De-grasping should not be imparted as a “technique” but offered as an existential demonstration: the therapist’s non-defensive, non-judgmental state of presence itself is the most powerful de-grasping intervention. This requires the therapist to integrate Jaspers’ empathic understanding (Einfühlung) with the Zen tradition of direct transmission from master to disciple, guiding the client not merely to “practice” mindfulness, but to “become” mindfulness within the relationship.

Although the transformative core of this work lies at a pre-reflective level, language, dialogue, and symbolic or aesthetic mediations (e.g., imagery, metaphor, or cinematic reference) may serve as supportive bridges, allowing lived experience to be gently articulated without being reduced to performance or explanation.

### 4.2 Theoretical inferences and practical implications

Based on the foregoing ontological analysis, it can be inferred that the awakening of death awareness does not necessarily increase anxiety; on the contrary, it can create the conditions for an authentic reconstruction of the self-schema by dismantling the foundation of the false self. At the phenomenological level, this process reveals a paradoxical truth: the narcissist’s self-schema, while appearing solid, is in fact a systematic denial of mortality, and is therefore inherently precarious precisely because mortality is an undeniable fact; whereas a healthy self-schema derives its resilience precisely from its fundamental acceptance of finitude. This inference points to a therapeutic direction that should not aim to enhance the narcissist’s “self-esteem,” but rather to assist them in reducing the compulsive need for their self-worth to be continually proven, allowing them to confront their inner barrenness, vulnerability, and inadequacy. Only when an individual no longer needs to experience the self as “exceptional” to ward off the fear of death can genuine self-exploration begin.

From the dimension of temporality, this inference implies: the narcissist’s temporal consciousness is colonized by the future—the present is merely a means to future glory; whereas the transformed temporal consciousness is open to the present—the future becomes an extension of the present, not its master. The congealing of temporality noted by Jaspers (19) is thus dissolved, allowing primary experience to be re-weighted in the “here-and-now,” forming a continuous stream of life no longer hijacked by defensive narratives. From an existentialist perspective, this process signifies the reclaiming of freedom: when an individual ceases to perform in response to death, choice becomes authentic choice, rather than a compulsive defensive reaction.

The above analysis further involves a reunderstanding of causality: trauma does not “cause” narcissism; rather, through the ontological rupture of shattered ontological security, it provides the possibility space for the emergence of narcissistic defenses. In other words, the pathogenicity of trauma lies not in the event itself, but in its destruction of the subject’s capacity to construct a stable world-picture. Without this collapse of the ontological foundation, similar trauma might lead to depression, anxiety, or other patterns. The clinical implication of this inference is that therapy should not over-focus on “repairing childhood memories,” but should strive to rebuild the continuity of the meaning system in the present. Even if childhood trauma is unchangeable, the individual can still re-endow it with new meaning through phenomenological reduction—shifting from “I was destroyed” to “I learned to perform survival amidst the fissures of being destroyed, and now I choose to learn authentic survival.”

The ontological breakthrough of this mediating model lies in its liberation of psychopathology from the cage of determinism. If one only emphasizes the causal power of childhood trauma, therapy falls into fatalism; by introducing ontological security as a mediator, it reveals the possibility of ontological choice: even given early failures, the individual can still re-choose, at the existential level, how to respond to their thrownness. This echoes the tension in Heidegger between “thrownness” (Geworfenheit) and “projection” (Entwurf)—we cannot choose the starting point of our thrownness, but we can choose how to project our being. Therefore, the ultimate goal of therapy is not to make the client “forget” the trauma, but to transform their relationship with it: from trauma as the ontological ground of self-definition, to trauma as a catalyst for authentic awakening.

The third layer of philosophical implication from the above analysis is this: the Zen practice of “non-self” (anātman) is not ethical preaching or cognitive correction, but a direct attack on the ontological core of narcissism through the persistent dismantling of the perceptual basis for a self-entity. Unlike existentialism, which emphasizes the “authentic self,” Zen holds that any self-construction, including the authentic self, carries potential for grasping. Therefore, the practice of de-grasping the self holds significant therapeutic value for the narcissist. It offers a path beyond constructing a healthy self—a more fundamental path of transcending self-concern. The fundamentality of this path lies in its suggestion that the ultimate possibility of healing narcissism is not “healthy narcissism,” but the loosening of self-grasping, to the point where the individual no longer regards self-worth as an entity requiring continual maintenance.

At the practical level, this inference means therapists must be wary of the trap inherent in the concept of “healthy narcissism.” If the therapeutic goal is set as helping the client establish “realistic and positive self-esteem,” it may merely replace the old grandiose self with a new, more socially adaptive idealized self (“I am recovered, I am healthy”), leaving the structure of self-grasping fundamentally unshaken. The Zen perspective suggests that genuine transformation occurs when the individual experiences the “non-apprehension of self, person, or beings”—in that moment, the need for specialness, the craving for moral privilege, and the tendency to instrumentalize others, having lost the grasping subject to which they adhere, naturally fall away. However, this “sudden awakening” (satori) is not a universal expected outcome of therapy, but rather a directional guide for ontological practice: every small step in therapy should be a movement towards “less grasping, more openness,” not towards the construction of a “better self-image.”

Ultimately, these three major inferences collectively point towards an integrative philosophical conclusion: the essence of narcissistic pathology is the alienation of the subject’s relationship with the world, and its possibility of healing lies in the restoration of relationality—not only the relationship with others (from exploitation to Being-with), but also the relationship with time (from future-colonization to present-openness), with death (from denial to assumption), and with language (from symbolic concealment to phenomenological description). Phenomenology provides the method of description, existentialism provides the courage for transformation, and Zen provides the wisdom for the loosening of grasping. The integration of these three opens a path for healing narcissism—this ancient yet modern psychological predicament—that transcends technicism and returns to the depths of existence.

## 5 Conclusion

This paper synthesizes psychoanalytic, cognitive-behavioral, neuroscientific, phenomenological, existential, and Buddhist accounts into the Existential Fracture model, arguing that NPD is not a constellation of surface traits but a structural rupture between primary lived experience and its symbolic integration. Ontological insecurity, triggered by mirroring failures and intensified by confrontation with boundary situations, compels the individual to replace the unstable lived self with a performative, idealized self whose sole purpose is to sustain the illusion of special exemption from finitude, temporality, and intersubjective vulnerability. Grandiose and vulnerable presentations are not separate disorders but functionally equivalent defensive choreographies: the former asserts exceptionality through overt superiority, the latter through moralized victimhood; both convert death anxiety into claims of privileged being and instrumentalize others as mirrors or threats to keep the fantasy intact. Empirical findings on brain activation, self-esteem defense, and cultural modulation are reinterpreted as downstream effects of this deeper representational blockage (45). The model thus unifies previously disparate data under a single explanatory arc: narcissistic symptoms are surface manifestations of a fractured world-picture that bars the individual from inhabiting the present, accepting thrownness, and experiencing non-instrumental relatedness.

Although the present model is primarily theoretical and phenomenological in orientation, it lends itself to empirical exploration through qualitative and mixed-methods approaches compatible with its underlying assumptions. For example, ontological insecurity may be investigated through in-depth phenomenological interviews focusing on lived temporality, embodiment, and meaning-making under conditions of narcissistic vulnerability. Similarly, the notion of the performative self may be operationalized via narrative analysis, examining recurrent themes of self-justification, future-oriented self-projection, and relational instrumentalization in clinical or autobiographical narratives. In addition, adapted clinical scales or interview-based measures could be developed to assess degrees of existential rigidity, performative self-relation, or openness to primary experience, provided that such instruments remain sensitive to contextual and experiential dimensions rather than reducing these constructs to decontextualized traits.

## Data Availability

The original contributions presented in the study are included in the article/supplementary material. Further inquiries can be directed to the corresponding author.
